# Gestational Dating by Urine Metabolic Profile at High Resolution Weekly Sampling Timepoints: Discovery and Validation

**DOI:** 10.3389/fmmed.2022.844280

**Published:** 2022-04-27

**Authors:** Karl G. Sylvester, Shiying Hao, Zhen Li, Zhi Han, Lu Tian, Subhashini Ladella, Ronald J. Wong, Gary M. Shaw, David K. Stevenson, Harvey J. Cohen, John C. Whitin, Doff B. McElhinney, Xuefeng B. Ling

**Affiliations:** ^1^ Department of Surgery, Stanford University School of Medicine, Stanford, CA, United States; ^2^ Department of Cardiothoracic Surgery, Stanford University School of Medicine, Stanford, CA, United States; ^3^ Clinical and Translational Research Program, Betty Irene Moore Children’s Heart Center, Lucile Packard Children’s Hospital, Palo Alto, CA, United States; ^4^ Department of Health Research and Policy, Stanford University, Stanford, CA, United States; ^5^ Department of Obstetrics and Gynecology, University of California San Francisco-Fresno, Fresno, Fresno, CA, United States; ^6^ Department of Pediatrics, Stanford University School of Medicine, Stanford, CA, United States

**Keywords:** normal pregnancy, gestational age, metabolite, urine, date of delivery

## Abstract

**Background:** Pregnancy triggers longitudinal metabolic alterations in women to allow precisely-programmed fetal growth. Comprehensive characterization of such a “metabolic clock” of pregnancy may provide a molecular reference in relation to studies of adverse pregnancy outcomes. However, a high-resolution temporal profile of metabolites along a healthy pregnancy remains to be defined.

**Methods:** Two independent, normal pregnancy cohorts with high-density weekly urine sampling (discovery: 478 samples from 19 subjects at California; validation: 171 samples from 10 subjects at Alabama) were studied. Urine samples were profiled by liquid chromatography-mass spectrometry (LC-MS) for untargeted metabolomics, which was applied for gestational age dating and prediction of time to delivery.

**Results:** 5,473 urinary metabolic features were identified. Partial least-squares discriminant analysis on features with robust signals (*n* = 1,716) revealed that the samples were distributed on the basis of the first two principal components according to their gestational age. Pathways of bile secretion, steroid hormone biosynthesis, pantohenate, and CoA biosynthesis, benzoate degradation, and phenylpropanoid biosynthesis were significantly regulated, which was collectively applied to discover and validate a predictive model that accurately captures the chronology of pregnancy. With six urine metabolites (acetylcholine, estriol-3-glucuronide, dehydroepiandrosterone sulfate, α-lactose, hydroxyexanoy-carnitine, and l-carnitine), models were constructed based on gradient-boosting decision trees to date gestational age in high accordance with ultrasound results, and to accurately predict time to delivery.

**Conclusion:** Our study characterizes the weekly baseline profile of the human pregnancy metabolome, which provides a high-resolution molecular reference for future studies of adverse pregnancy outcomes.

## Introduction

The accurate assessment of gestational age (GA) during the prenatal period is critically important (Butt et al., 2014; Unger et al., 2019). Pregnancy dating with high precision can optimize the clinical benefits of prenatal screening tests (Wald et al., 1992; Rahim et al., 2002) and identify early signs of pregnancy complications (Smith et al., 1998; Kallen, 2004; Fung et al., 2020). First trimester ultrasound imaging measurement is considered as the standard of care to determine GA and date of delivery, and it has been a routine practice recommended to every pregnant woman (Taipale and Hiilesmaa, 2001; Butt et al., 2014). The accuracy of ultrasound reduces as pregnancy progresses because fetal growth becomes more variable, leading to a higher likelihood of misclassification (Caughey et al., 2008). To address this, the GA calculation formula was modified to reduce errors and biases of ultrasound estimation at late gestation, and date of last menstrual period (LMP) was used in combination with ultrasound for improved accuracy (Butt et al., 2014). However, these approaches are subject to clinical justification and accurate recall of cycle characteristics (Rosenberg et al., 2009). Moreover, the high costs of ultrasound and the fact that its accuracy can be affected by operator variability, and its availability in disadvantaged areas, especially in lower- and middle-income countries where poor attendance of pregnant women to the first prenatal visit remains an issue (Jehan et al., 2010; Moore et al., 2015).

Alternative methods have been developed to assess GA dating. Recent studies have shown that a few molecular signatures such as genes, proteins, and metabolites in maternal blood and urine are associated with fetal growth (Wright et al., 2015; Maitre et al., 2016; Aghaeepour et al., 2017; Jiang et al., 2017; Serpero et al., 2017; Zhang et al., 2017; Aghaeepour et al., 2018; Ngo et al., 2018; Ghaemi et al., 2019; Han et al., 2019; Hao et al., 2020; Liang et al., 2020; Sylvester et al., 2020; Ghaemi et al., 2021; Tarca et al., 2021). Algorithms of GA estimation and date of delivery prediction were developed and validated using omics data measured in maternal blood or urine samples collected longitudinally from the first to the third trimesters (Aghaeepour et al., 2017; Aghaeepour et al., 2018; Ngo et al., 2018; Hao et al., 2020; Liang et al., 2020; Sylvester et al., 2020; Ghaemi et al., 2021). Such findings have revealed the potential of using noninvasive, low-cost, rapid tests as alternative methods of pregnancy dating, especially in low-resource settings where ultrasound measurements are unreliable and cost-prohibitive.

Compared with blood, the noninvasive nature of urine collection allows for convenient weekly sampling. In this study, we hypothesized that longitudinal urine metabolic profiling of pregnancy reflects the temporal progression of fetal development with a high degree of precision. We used urine samples collected weekly from early to late gestation and the postpartum period from two cohorts of normal, full-term pregnant women. The two cohorts were independently assembled in two different states in the US. Using liquid chromatography-mass spectrometry (LC-MS)-based untargeted metabolomics, we identified a panel of metabolic compounds and pathways that were highly associated with GA measured by ultrasound in the first-trimester. We developed a model to estimate GA using a set of annotated metabolites with samples from one cohort and validated it on the other cohort. We also developed and validated a model to predict time to delivery. Our findings suggest that modeling of metabolic profiling in maternal urine may serve as a noninvasive, cost-effective, and robust approach to GA dating and date of delivery prediction.

## Materials and Methods

### Study Population and Sample Collection

Pregnant women were enrolled between September 2013 and May 2016 at Stanford University, California (California cohort) and between December 2013 and February 2016 at the University of Alabama, Alabama (Alabama cohort). Urine samples were collected weekly from week 9 until delivery for women in the California cohort and week 15 until delivery for subjects in the Alabama cohort, and one sample collected postpartum. Dates of sampling and delivery were documented. Clinical information was collected. Ultrasound measurements at the first trimester were recorded. Women with normal, full-term pregnancies were included in the study cohorts. A normal, full-term pregnancy refers to a pregnancy at 37–41 weeks and without known complications. The study was approved by ethics committees at Stanford University and the University of Alabama, and written informed consents were obtained from all participants.

### Study Design

The study was conducted in two phases: 1) modeling to devise a metabolite-based estimation of GA during normal, full-term pregnancies; and 2) modeling to devise a metabolic panel predictive of time to delivery. In this study, the “gold” standard of GA was the ultrasound measurement based on the crown-rump length at the first trimester (Robinson, 1973). Metabolic concentrations in each urine sample were measured by global mass spectrometry (MS) analysis. Models that estimated GA and predicted time to delivery were developed using the California cohort and validated using the Alabama cohort. All statistical analyses were done in R software.

### Urine Metabolite Extraction

The urinary metabolites were extracted using a protein precipitation-based approach. Briefly, 10 µL of urine sample was extracted with 100 µL of methanol containing 5 μg/ml of ^13^C_5_, ^15^N-l-proline^, 13^C_6_-l-arginine, and D_5_-l-glutamine. These externally spiked exogenous isotope-labelled metabolites were used as references for sample preparation and extraction efficiency. The extract was then vortexed for 1 minute and centrifuged at 12,000 × g for 5 min 90 µL of supernatant was then collected for the global metabolomics analysis.

### Global Metabolomics Analysis

Following sample preparation, 10 µl of extract was injected onto a ZIC-HILIC column (2.1 mm × 100 mm × 3.5 μm) from EMD Millipore (Burlington, MA). The chromatographic separation was carried out using 10-mM ammonium acetate in water (A) and 10-mM ammonium acetate in acetonitrile (B) as mobile phases. The urine metabolites were eluted off the column using a linear gradient of 10–90% phase B over 7.5 min at 0.4 ml/min, and the column was re-equilibrated at 10% phase B from 7.6 to 10 min at 0.5 ml/min before the next injection. The column oven and autosampler temperatures were maintained at 40 and 4°C, respectively, throughout the analysis.

The data was acquired by a Q Exactive plus mass spectrometer in both electrospray positive and negative modes *via* a data-dependent manner. The source conditions were set at 3.5/−3.5 kV for Spray Voltages, 300°C for Vaporizer Temp, 300°C for Capillary Temp, 55.0% for S-Lens, 40 for Sheath Gas, 10 for Auxiliary Gas, and 0 for Sweep Gas. The spectra were acquired using following parameters: automatic gain control (AGC, MS1) = 1 × 10^6^, AGC (MS2) = 1 × 10^5^, Injection Time (MS1) = 100 m, Injection Time (MS2) = 50 m, Mass Range (MS) = 70–1,000 Da (Da), Resolution (MS1) = 70,000, Resolution (MS2) = 17,500, data dependent top-5 experiment (MS2) data acquisision, Isolation Window (MS2) = 1.0 Da, Dynamic Exclusion = 14 s, and Collision Energy (CE) = 25 eV. Between batch injections, the instrument was calibrated using external standards in both positive and negative modes to ensure a mass accuracy of less than one part per million (ppm).

### Quality Control

A set of urine samples were collected longitudinally from a healthy volunteer across gestation, and the longitudinal urine samples were pooled to generate a urine sample for quality control (QC) purposes. Prior to the batch analysis, five replicates of QC urine extract were injected into the system for proper conditioning. Along the batch analysis, QC urine samples were analyzed repetitively at a frequency of one QC injection per 10 testing samples to allow the systematic assessment of the data quality. In addition, the signal responses of spiked internal standards (ISs) were checked batchwise to evaluate for the quality of metabolite extraction and sample preparation.

### Data Pre-Processing

The detected metabolic features were extracted, aligned, integrated, grouped, and annotated using the XCMS package in R to obtain a matrix of metabolite intensities versus samples. The obtained features were normalized by a QC-based robust locally estimated scatterplot smoothing (LOESS) signal correction approach, and each feature was independently corrected by fitting a LOESS curve to the signal response measured in QC replicates injected repeatedly along the batch (Dunn et al., 2011). Features with coefficient variation (CV) ≤ 20% in QC and missing values in ≤30% of samples were selected for downstream analysis. After missing value imputation, the normalized intensities of qualified metabolites were divided by the creatinine values in a sample-wise manner to correct for the differential dilutions of urine samples.

### Statistical Analyses

Partial least-squares discriminant analysis (PLS-DA) was performed to characterize metabolic profiling of samples collected at the first, second, third trimesters and the postpartum period. The correlation coefficients of detected metabolites to GA were determined by the Pearson approach. A global false discovery rate (FDR) of 5% was applied to correct for the errors from multiple hypothesis testing. Correlations between the abundances of metabolites that were significantly associated with GA (Peasron’s r > 0.3 or r < −0.3) were presented using a two-dimensional self-organizing map (SOM), where metabolites were categorized into clusters with similar abundance patterns. Prior to the modeling process, data preprocess (average with 3 weeks window; 40 weeks). All statistical analyses were preformed using R packages (Heinemann, 2019).

### Metabolite Pathway Analyses

The metabolic features were identified by matching their accurate precursor masses against a local database compiling a variety of public databases such as HMDB and MS-DIAL by using a tolerance of ± 5 ppm. Upon matching, the assigned metabolite identities were further validated by the retention time and fragmentation pattern from our local library containing 600 + authentic standards that were analyzed under the identical condition. The significant metabolites with validated identities were mapped to known *Homo sapiens* pathways in Kyoto Encyclopedia of Genes and Genomes (KEGG) database to characterize the pertaining pathway network and identify the meaningful pathways in the course of pregnancy development. Metabolic pathway-based pregnancy modeling was implemented as previously described (Sylvester et al., 2020) to estimate GA and time to delivery. Models were derived with the samples from the California cohort, and validated with the samples from the Alabama cohort. A tree based gradient boosting algorithm was utilized to construct the models (Chen and Guestrin, 2016). Model performance was assessed by Pearson’s correlation between model estimation and ultrasound results (for the GA dating model), and between model prediction and observed values (for the time-to-delivery predictive model). Errors were calculated with samples collected at the second trimester, the third trimester, and the combined period, respectively, and aggregated by subjects. Profiles of specific metabolites in urine were compared with those in sera.

### Metabolite Structure Determination and Compound-Based Modeling

Metabolite biomarker identification was performed as a Tier 1 or 2 identification with chemical standards according to MSI (Viant et al., 2017). With tandem mass spectrometry (MS/MS, Thermo Q Exactive plus) data of urine samples and manual review confirmation, the generated MS1/MS2 pairs were searched in the public databases: HMDB (http://www.hmdb.ca/), MoNA (http://mona.fiehnlab.ucdavis.edu/), MassBank (http://www.massbank.jp/), METLIN (https://metlin.scripps.edu), and NIST (https://www.nist.gov/). The metabolites of interest were procured and subjected to a Tier one identification comparing the retention time, MS1 and MS2 patterns with the biomarker candidates, using the same LCMS/MS protocol with the sample analysis.

### Explainability of the Predictive Models

To allow direct insight into the model, we implemented an “explainer”, based on Shapley values (Lundberg et al., 2020) from game theory, to explain the association between the input and predicted output. Our tree-based machine learning models such as random forests, decision trees, and gradient boosted trees are popular non-linear predictive models, yet the interpretability of tree-based models is low. Shapley value approach combines many high-quality local explanations to allow us to represent global structure while retaining local faithfulness to the original model. Shapley values used for explanations of the model were calculated with the SHAP package (https://github.com/slundberg/shap).

### Modelling Scenarios

To investigate model performance between different machine learning approaches, we conducted comparative modelling with XGBoost, random forest, and elastic net classifiers for all combinations of predictive features and cohorts.

## Results

### Baseline Characteristics

**FIGURE 1:**
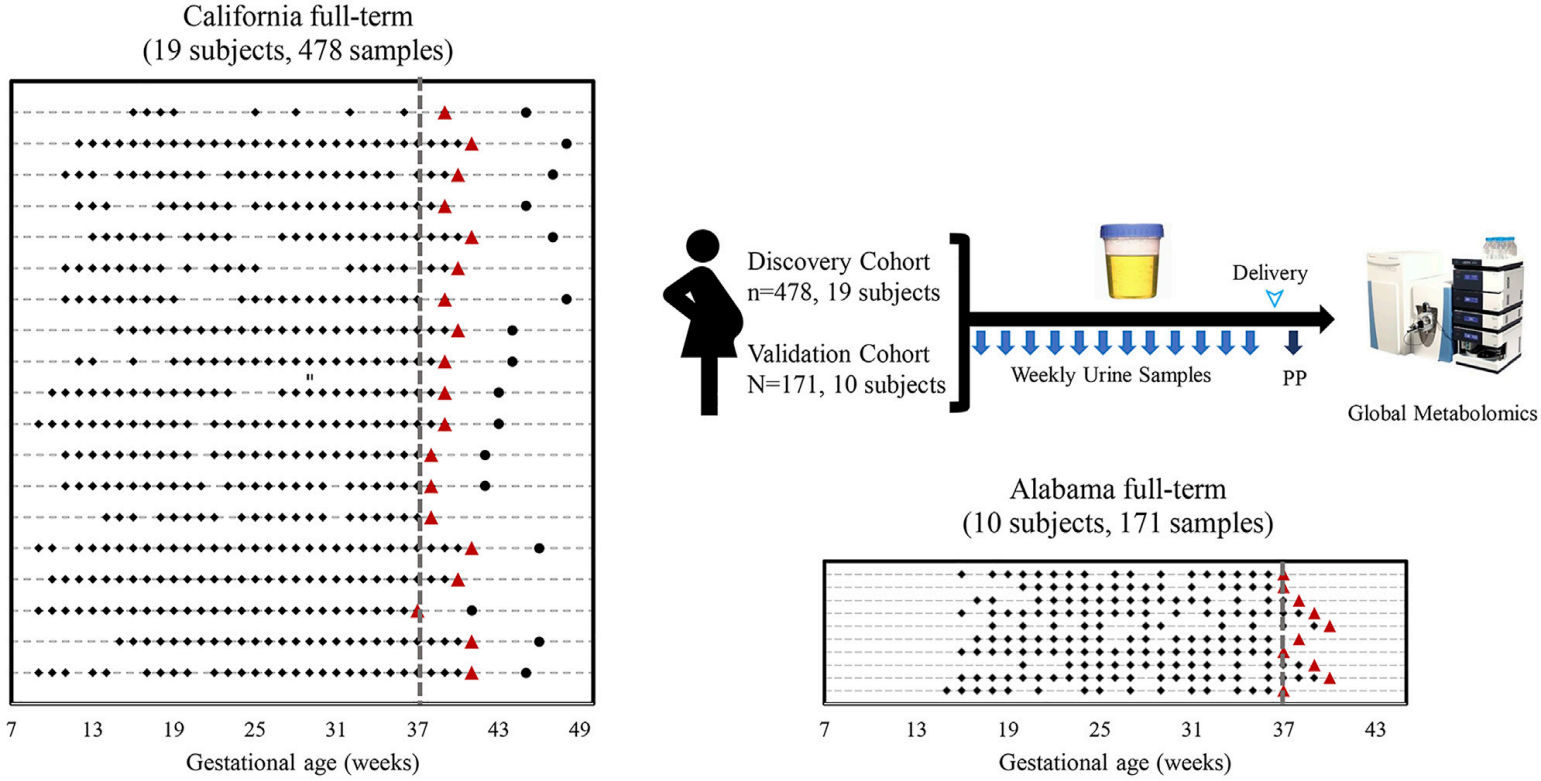
Sampling scheme and study cohorts. Sample collection timelines from the California cohort and the Alabama cohort. Diamonds, circles, triangles and lines indicate sample collection times before delivery, sample collection times after delivery, delivery dates, and individual woman, respectively.

As shown in Figure 1, the California cohort consisted of 19 full-term pregnancies with 478 urine samples, and the Alabama cohort consisted of 10 full-term pregnancies with 171 urine samples. Each subject had 9 to 32 samples collected prior to delivery and one sample collected postpartum. Demographics of the two cohorts are shown in Supplementary Table S1 and S2. All the subjects in the California cohort were white while all the subjects in the Alabama cohort were black. Compared with subjects in the California cohort, subjects in the Alabama cohort were younger (*p* < 0.001: 32.2 vs. 26). The average gestational duration of California subjects is 39.5 weeks, longer than the Alabama moms’ (38.2 weeks). Compared with subjects in the California cohort, subjects in the Alabama cohort had higher pre-pregnancy body mass index (BMI; *p* = 0.01). The average BMI of California subjects is 21.8, ranging from 20.2 to 24.7, while that of Alabama subjects is 29.8, ranging from 26.6 to 32.5. All the Alabama subjects had previous pregnancies, while 47% (9 of 19) of the California subjects were in the first pregnancies.

### A Unique Pregnancy Progression Pattern Revealed by Weekly Sampled Urine Metabolites

**FIGURE 2:**
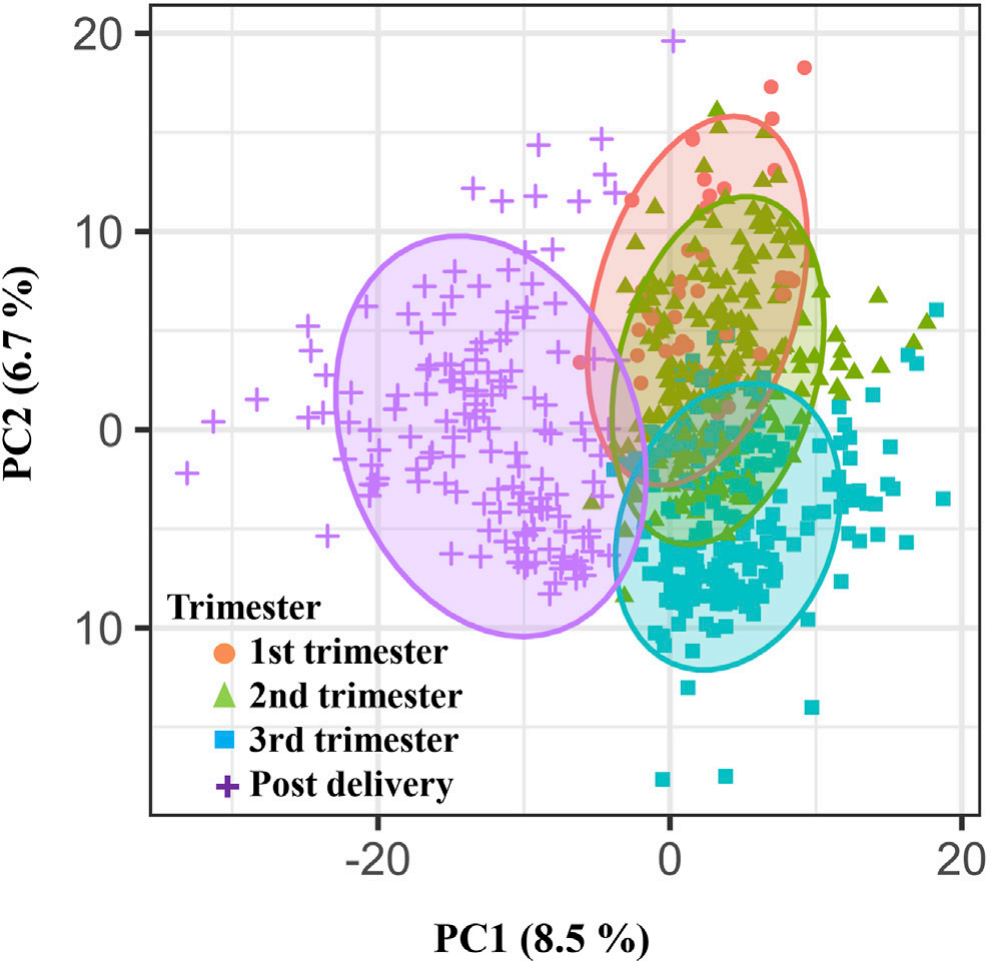
Weekly pregnancy progression with a unique pattern ordered by urine metabolites. Distribution of individual samples in partial least-squares discriminant analysis (PLS-DA) based on 1716 urinary metabolic features (840 positive and 876 negative features) as a function of pregnancy stages. The two orthogonal components with most of the inertia are shown.

A total of 5,473 metabolic features were identified by LC-MS-based global metabolomic profiling of samples in the California cohort (Supplymentary Figure S1). Of these, 1,716 features were selected with a missing value percentage less than 30% across all samples. These features were examined globally with PLS-DA (Figure 2), revealing a clustering pattern of ordered pregnancy progression as a function of the GAs in the first/second/third trimesters and the postpartum period.

### Identification of Urine Metabolites Associated With GA

**FIGURE 3:**
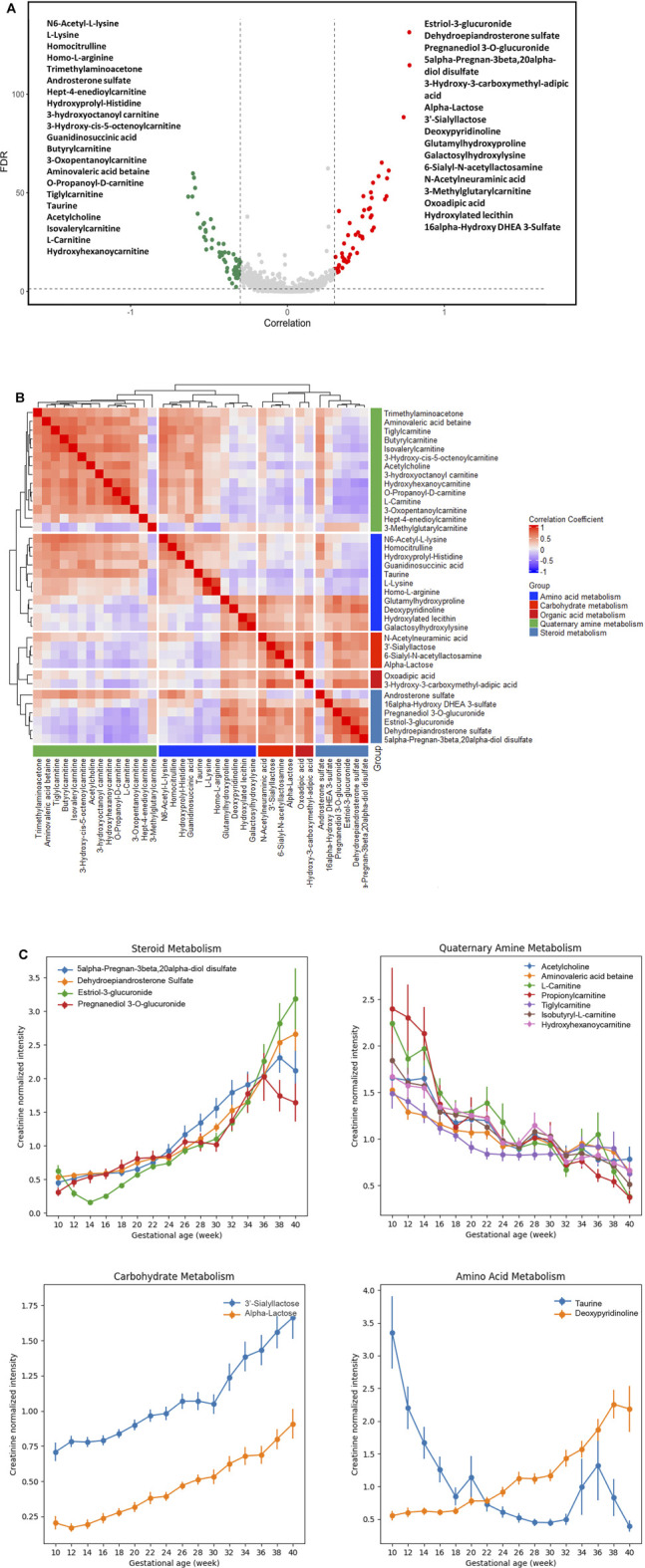
Urine metabolites of differential GA correlation and abundance during normal pregnancy progression. **(A)** The vertical axis (*y*-axis) displays the −log10 (FDR-adjusted *p*) with features altered during pregnancy gestational age, and the horizontal axis (*x*-axis) displays the Pearson correlation coefficients between features and gestational ages. The red dots represent features positively correlated with gestational ages; the green dots represent features negatively correlated with gestational ages. Names of the most significant 37 metabolites were listed (with MS level 1 or 2 identification). **(B)** Heatmap of Pearson correlation coefficients between the expressions of the 37 metabolites that were significantly associated with gestational ages. The significant urinary metabolites were classified into five clusters for the ease of visualization. **(C)** Level changes in metabolites associated with steroid metabolism, quaternary amine metabolism, carbohydrate metabolism, and amino acid metabolism as a function of gestational age. Values were smoothed in a moving window of 3 weeks. The intensities are shown as mean ± SEM (standard error of the mean).

A process of metabolite feature reduction was performed using the workflow in Supplymentary Figure S1. 885 features were found to be significantly correlated to gestations with a FDR [Benjamin and Hochberg method (Hochberg and Benjamini, 1990)] < 0.05, suggesting extensive urine metabolic changes occurred during the pregnancy progression. Filtered by an additional GA correlation criterion (Pearson’s r > 0.3 or < −0.3), a total of 119 metabolites were identified by LCMS profiling. 37/119 compound identities (Figure 3A) were determined by LC-MS/MS and compound library annotation analyses.

### Co-Regulation Relationships Among Functional Group Metabolites

We analyzed the correlation in metabolite abundance along GA by characterizing and clustering the top 37 annotated metabolites. Based upon the existing structural and biological annotations, these 37 metabolites were categorized/clustered into five groups (Figure 3B): amino acid, carbohydrate, organic acid, quaternary amine, and steroid metabolisms.

The largest cluster was composed of various carnitine metabolites. These metabolites are indispensable for the transport of activated long-chain fatty acids from the cytosol to the mitochondrial matrix, where β-oxidation occurs, and the transfer of products of peroxisomal β-oxidation to the mitochondria for oxidation in the citrate cycle, the modulation of the acyl-coenzyme A (CoA)/CoA-ratio and the storage of energy as acetylcarnitine (McGarry and Brown, 1997; Rebouche and Seim, 1998; Steiber et al., 2004). We found that their abundance decreased along GAs (Figure 3C). The gestational patterns of the carnitine metabolites in maternal urine were similar to those in maternal plasma (Bargen-Lockner et al., 1981; Keller et al., 2009), which markedly declined to about half of the concentrations of non-pregnant women.

Positively-correlated patterns were observed between carnitine and basic amino acid clusters (Figure 3B). Such findings are in line with the fact that the endogenous biosynthesis of carnitine involves a complex series of reactions with lysine providing the carbon backbone (Keller et al., 2009). In the amino acid cluster, deoxypyridinoline had a positive correlation with GA, while taurine had a negative correlation with GA (Figure 3C).

Positively correlated patterns were also observed between steroid and carbohydrate metabolisms (Figure 3B). Metabolites associated with these two clusters, including 5-alpha-pregnan-3beta, 20alpha-diol disulfate, dehydroepiandrosterone sulfate (DHEA-S), estriol-3-glucuronide, pregnanediol 3-O-glucuronide, 3′-sialyllactose, and α-lactose, were all positively correlated with GA (Figure 3C).

### Metabolic Pathway Adaptions Are Associated With GA

**FIGURE 4:**
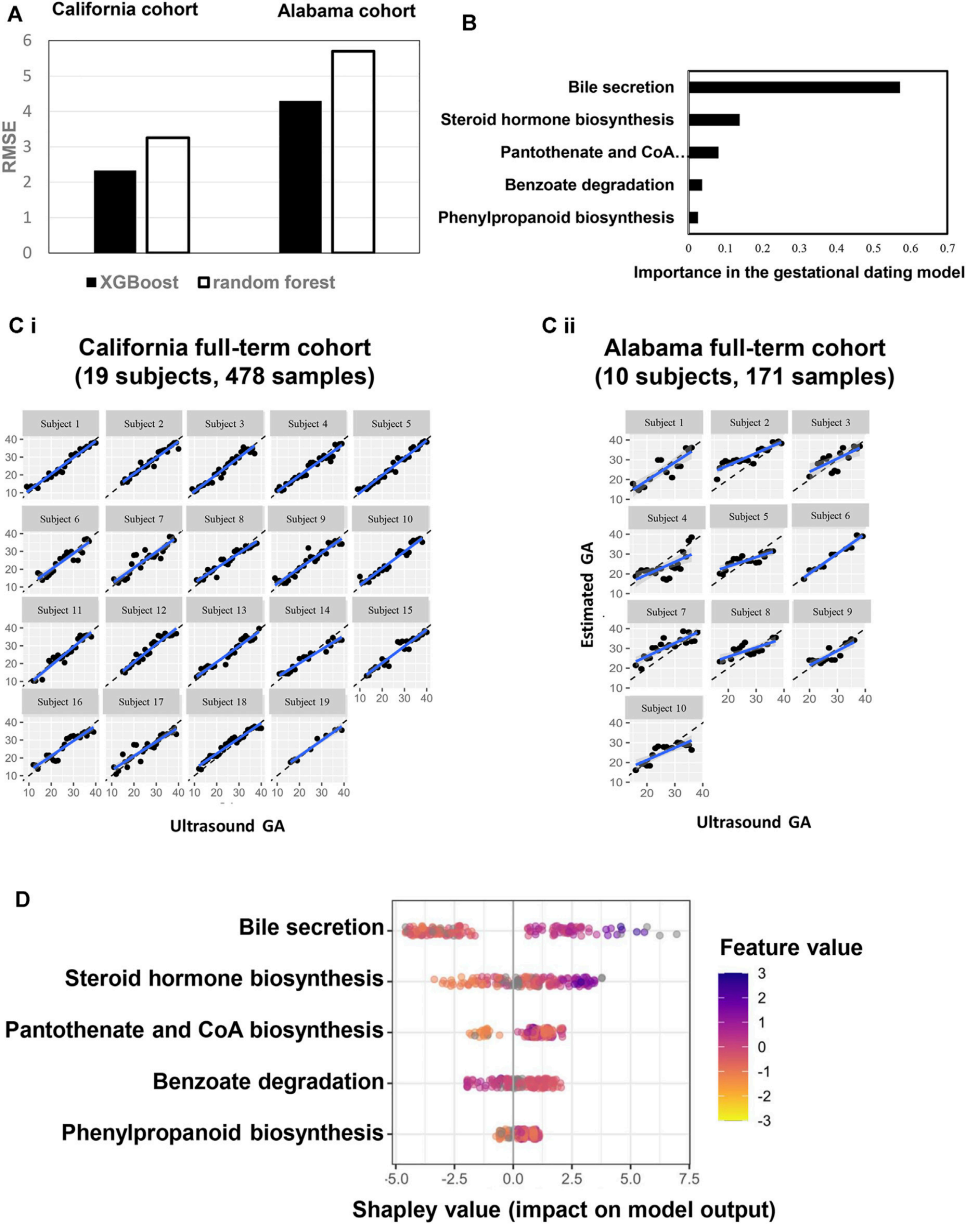
A pathway-based model to estimate GA. **(A)** performance comparison between two machine learning approaches (XGBoost: maximum depth of a tree is four, step size shrinkage (eta) is 0.3, and number of iteration is 19; random forest: number of tree is three, step size shrinkage (eta) is 0.3). RMSE were calculated to quantify and compare the predictive effectiveness between California and Alabama cohorts. **(B)** Importance of each pathway in the model. **(C)** Highly correlated patterns between GA estimated by the ultrasound (*x* axis) and GA estimated by the pathway-based model (*Y* axis) for each subject. Left: California cohort. Right: Alabama cohort. GA: gestational age. **(D)** Each row represents a significant pathway, and each point is the Shapley value of an sample. Redder sample points indicate that the value of the pathway activity is larger, and yellower sample points indicate that the value of the pathway activity is smaller; the abscissae represent the Shapley values.

The 37 annotated metabolites were mapped to five pathways: bile secretion, steroid hormone biosynthesis, pantothenate and CoA biosyntheses, benzoate degradation, and phenylpropanoid biosynthesis. Metabolic-pathway-based GA dating models was constructed based on the five pathways, using XGBoost and random forest approaches (Figure 4A). XGBoost outperforming random forest (Figure 4A), bile secretion was the most important feature in the XGBoost modeling (Figure 4B). Model performance was assessed with each cohort (California cohort: Pearson’s R = 0.96; Alabama cohort: Pearson’s R = 0.76; Supplymentary Figure S2) and with each subject [California cohort: Pearson’s R = 0.97 (0.96, 0.98); Alabama cohort: Pearson’s R = 0.84 (0.77, 0.87); Figure 4C]. Summary plot of Shapley values computed for each patient individually in the test partition. The pathways are sorted top-down based on their global contribution which is in line with Figure 4D. The distance of a dot representing a sample from the vertical line indicates its contribution. The color of a dot indicates feature value for that sample. Blue and pink color represent extreme values of the feature. Shapley values on the right side of vertical axes “push” predictions towards the high GA correlation and those on the left side towards the low correlation.

### Six Metabolic Markers Predict GA

**FIGURE 5:**
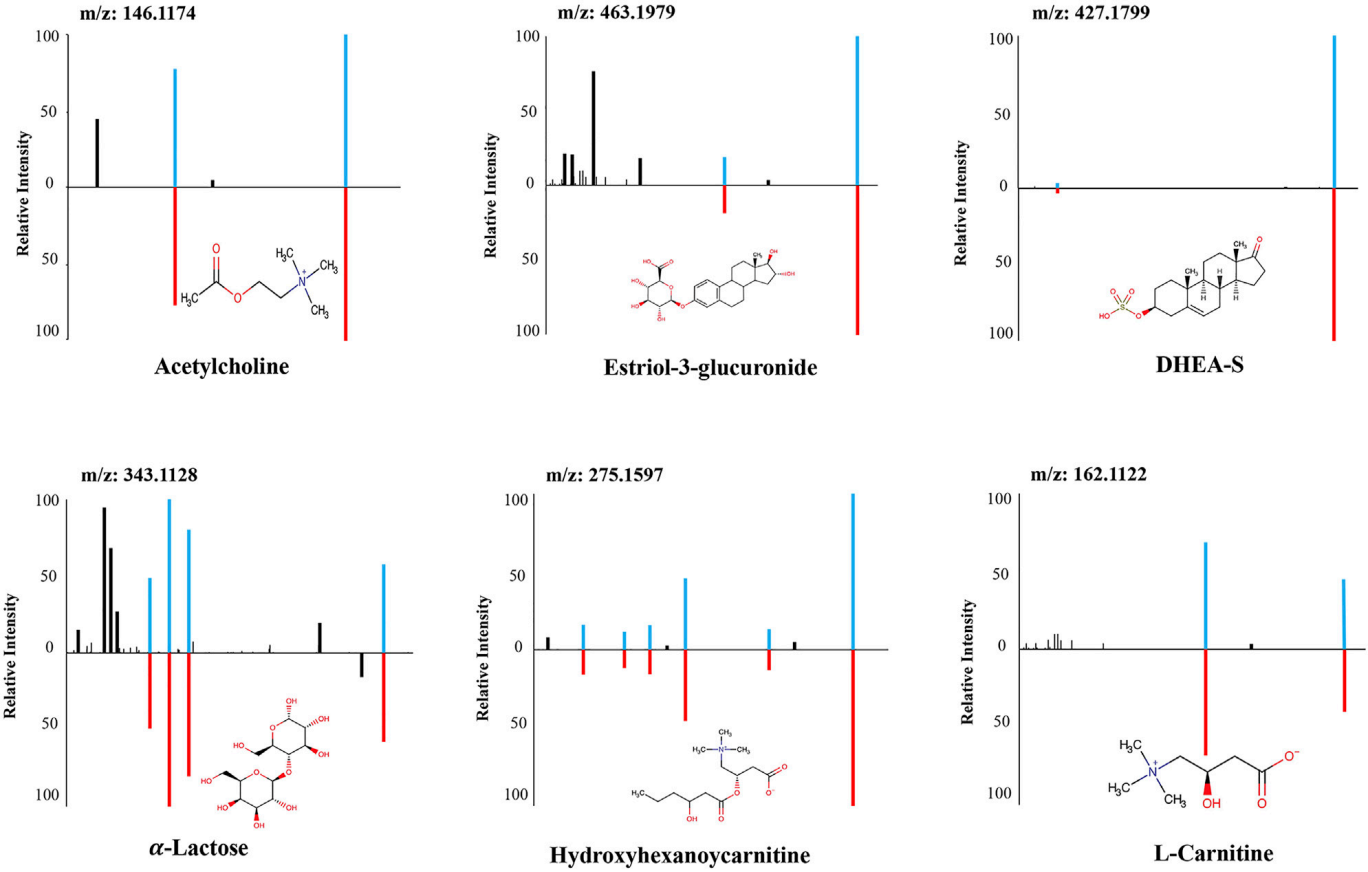
The structural identification of the six metabolites by MS/MS fragmentation against authentic standards. MS/MS: Tandem mass spectrometry.

**FIGURE 6:**
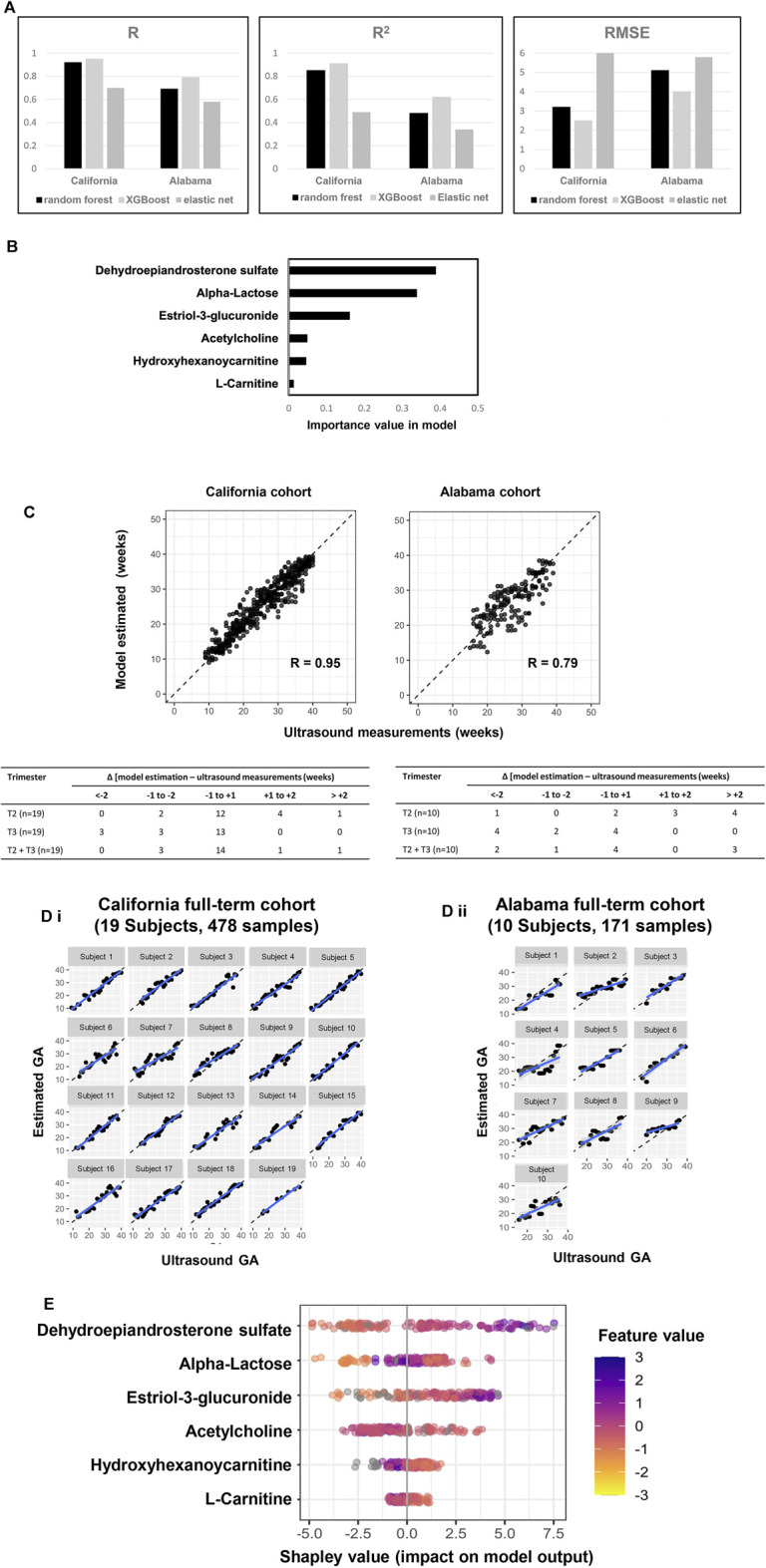
A six-metabolite-based model to estimate GA. **(A)** Performance comparison across three machine learning approaches (XGBoost: maximum depth of a tree is five, step size shrinkage (eta) is 0.3, number of iteration is 19; random forest: number of tree is two, step size shrinkage (eta) is 0.3; and elastic net: alpha is 0.26, lambda minimum is 0.06). R, *R* ^2^ and RMSE of chronology pregnancy age are calculated in both California and Alabama cohorts. **(B)** Importance of each metabolite in the model. **(C)** Top: GA estimated by the model versus GA measured by ultrasound at the California cohort (left) and the Alabama cohort (right). Pearson’s r at each cohort was calculated. Bottom: Distribution of the differences between the model estimation and the ultrasound measurements at the California cohort (left) and the Alabama cohort (right). Differences were calculated as the mean difference values in samples collected at the second trimester (T2), the third trimester (T3), and the second and third trimesters (T2 + T3) associated with a subject. **(D)** Highly correlated patterns between GA estimated by the ultrasound (*x* axis) and GA estimated by the six-metabolite-based model (*Y* axis) for each subject. Left: California cohort. Right: Alabama cohort. **(E)** Each row represents a significant metabolite, and each point is the Shapley value of an sample. Redder sample points indicate that the value of the metabolite activity is larger, and yellower sample points indicate that the value of the metabolite activity is smaller; the abscissae represent the Shapley values.

Six metabolic marker compounds of pregnancy GA dating were procured and subjected to a Tier 1 identification comparing the retention time, MS1 and MS2 patterns with the biomarker candidates, using the same LCMS/MS protocol with the urine sample analysis: acetylcholine, estriol-3-glucuronide, DHEA-S, α-lactose, hydroxyhexanoycarnitine, and l-carnitine (Figure 5). GA dating models were constructed based on the six metabolic markers, using XGBoost, random forest and elastic net approaches (Figure 6A). XGBoost outperforming random forest (Figure 6A, R, *R* ^2^, and RMSE analysis), DHEA-S and α-lactose were two most important features in XGBoost modelling (Figure 6B). Model performance was assessed with each cohort (California cohort: Pearson’s R = 0.95; Alabama cohort: Pearson’s R = 0.79; Figure 6C) and with each subject [California cohort: Pearson’s R = 0.97 (0.96, 0.98); Alabama cohort: Pearson’s r = 0.87 (0.82, 0.92); Figure 6D]. Differences between the model estimation and ultrasound results were calculated (Figure 6C). There were 73.7% (14 of 19 subjects) of the estimates in California cohort and 40.0% (4 of 10 subjects) of the estimates in Alabama cohort within ±1 week of the ultrasound results. Summary plot (Figure 6E) of Shapley values computed for each patient individually in the test partition. The metabolite biomarkers are sorted top-down based on their global contribution which is in line with Figure 6B. The distance of a dot representing a sample from the vertical line indicates its contribution. The color of a dot indicates feature value for that sample. Blue and pink color represent extreme values of the feature. Shapley values on the right side of vertical axes “push” predictions towards the high GA correlation and those on the left side towards the low correlation.

### Six Metabolic Biomarker Panel Predicts Time to Delivery

**FIGURE 7:**
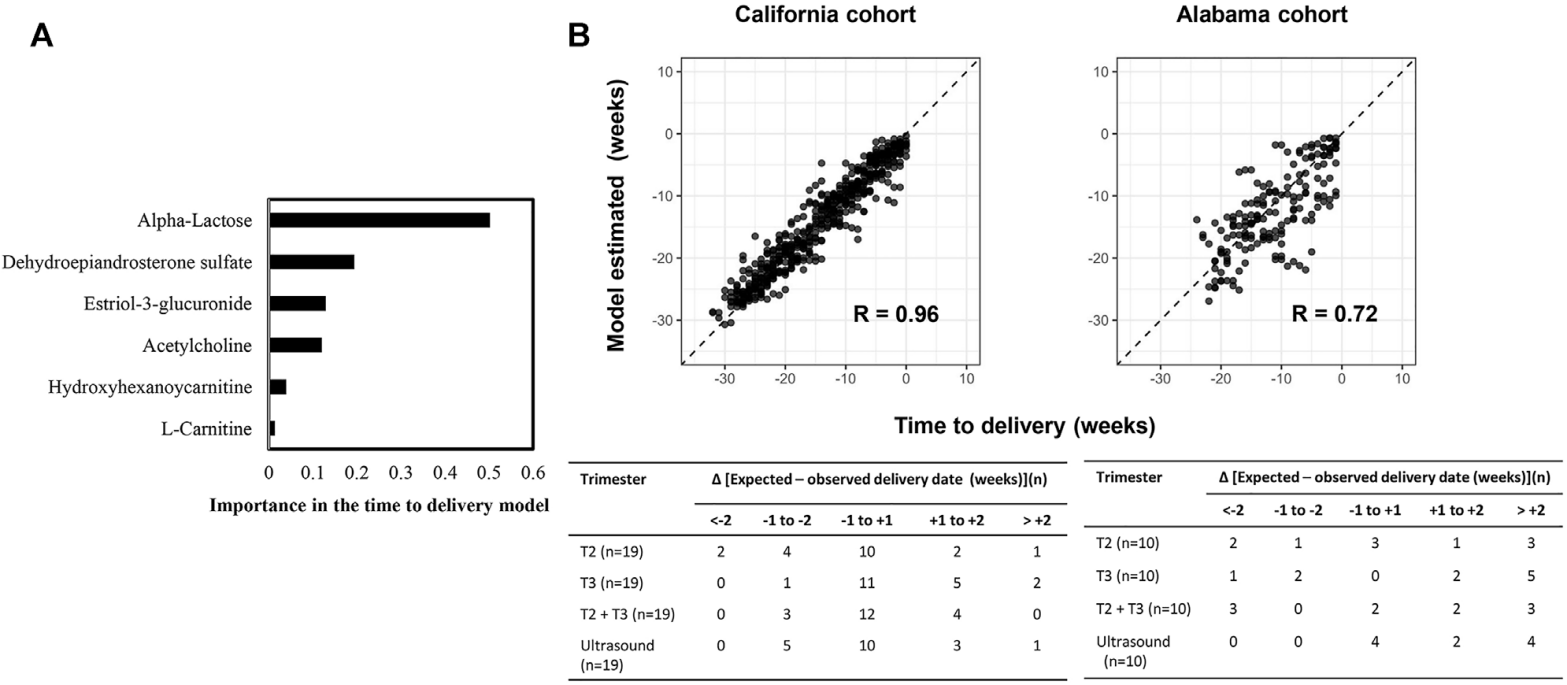
A six-metabolite-based model to predict time to delivery. **(A)** Importance of each metabolite in the model. **(B)** Top: Time to delivery predicted by the model versus observed time to delivery at the California cohort (left) and the Alabama cohort (right). Pearson’s r at each cohort was calculated. Bottom: Distribution of the differences between the delivery dates predicted by the model or ultrasound and the observed delivery dates at the California cohort (left) and the Alabama cohort (right). Differences were calculated as the mean difference values in samples collected at the second trimester (T2), the third trimester (T3), and the second and third trimesters (T2+T3) associated with a subject.

We developed and validated a separate model predictive of time to delivery using the 6 GA dating markers. Like the GA dating model, DHEA-S and α-lactose were top features in predicting time to delivery (Figure 7A). In the second trimester, 52.6% (10 of 19) of the prediction in the California cohort and 30.0% (3 of 10) of the prediction in the Alabama cohort had errors of less than 1 week, which was similar to the performance of the expectation by the first-trimester ultrasound results (California: *p* = 0.9; Alabama: *p* = 0.2; Figure 7B).

## Discussion

In this study, we enrolled pregnant women from two sites in the US, collected their urine samples on a weekly basis from the first to the third trimesters, and identified urinary metabolites associated with fetal growth during a normal, full-term pregnancy. Among the 5,473 metabolites obtained from raw MS signal, we identified 37 annotated metabolites associated with GA. These 37 metabolites are associated with five pathways, with which we built a pathway-based GA dating model. We further selected six metabolites to construct two metabolite-based models to estimate GA and time to delivery, respectively. In the validation, 40% of the GA estimates at the second and the third trimesters fell in ±1 week of the first-trimester ultrasound measurements, and 30% of the time-to-delivery predictions at the second trimester had differences of less then 1 week from the real values. These results validated the study hypothesis that longitudinal analysis of maternal metabolic profiling in urine could establish a metabolic clock for normal gestational development and enable a direct metabolic approach to determine expected delivery dates with comparable accuracy to ultrasound, creating the basis for an non-invasive gestational dating method.

The study revealed the clinical utility of urine metabolic profiling of pregnant women. GA and time to delivery can be predicted by a panel of six metabolites; the panel can be translated into a noninvasive urine test product. It provides an alternative option to measure the fetal growth, which will benefit women who have limited access to ultrasound.

The study characterized a baseline metabolic profile with a resolution down to 1 week. The findings were supported by the large sample size and the high-density cohort, resulting in a high statistical power and a high percentage of identifying true patterns. It allowed to detect changes in body both by weeks and by trimesters, which can help to precisely locate transition points of metabolites and pathways, during a normal full-term pregnancy. To our knowledge, it was the first study on pregnant women using urine samples collected weekly from the first trimester to delivery.

Deviation from the baseline metabolic profile established in this study can be an indicator of an abnormal pregnancy that leads to adverse outcomes. Two most important metabolite markers in the GA dating model, DHEA-S, and α-lactose, were found in association with preterm birth and gestational diabetes mellitus (Sachse et al., 2012; Sundararajan et al., 2021; Tian et al., 2021). Alterations in concentrations of the two markers may lead to detectable differences between the model estimation and the ultrasound results, which can be utilized to diagnose and predict adverse outcomes.

Pathways identified in this study were also reported by previous studies on pregnancy. Bile acids increase from early to late gestation (Gagnon et al., 2021). Steroid hormones are involved in placental development (Solano and Arck, 2019; Canumil et al., 2021). The pantothenate and CoA biosynthesis pathway may be associated with nutritional status in pregnancy (Bowman et al., 2019). Benzoate degradation are highly enriched in the placenta (Gomez-Arango et al., 2017). The phenylpropanoid biosynthesis pathway are enriched in women with gestational diabetes mellitus compared with those with a healthy pregnancy (Meng et al., 2021).

There seems to be no standardized normalization method for analyzing the urinary biomarkers, as some studies normalize with urinary creatinine (uCr), urine volume (uVol), or leave biomarker un-normalized. Cr is a 0.13 kD end product of muscle catabolism and usually produced at a fairly constant rate by the body (National Kidney Foundation, 2002; Levey et al., 2014a; Levey et al., 2014b). It is nonprotein bound and freely filtered and excreted in urine. We observed that raw intensity of uCr was decreased from the second to the third trimester (Supplymentary Figure S4). The uCr normalization is important and effective. The positive association between the urine DHEA-S and GA was increased after normalization (raw: Pearson’s r = 0.57; normalized: Pearson’s r = 0.76; *p* < 0.0001).

We also measured the urinary metabolic markers in sera from the 19 pregnant women in California cohort (Supplymentary Figure S3) and our results are in line with previous finding (Liang et al., 2020; Sylvester et al., 2020). Raw intensity and creatinine-normalized DHEA-S in urine were both positively associated with GA (Figure 3A and Supplymentary Figure S4), whereas it decreased in sera from the first to the third trimesters (Pearson’s r = −0.83, *p* < 0.0001).

**FIGURE 8:**
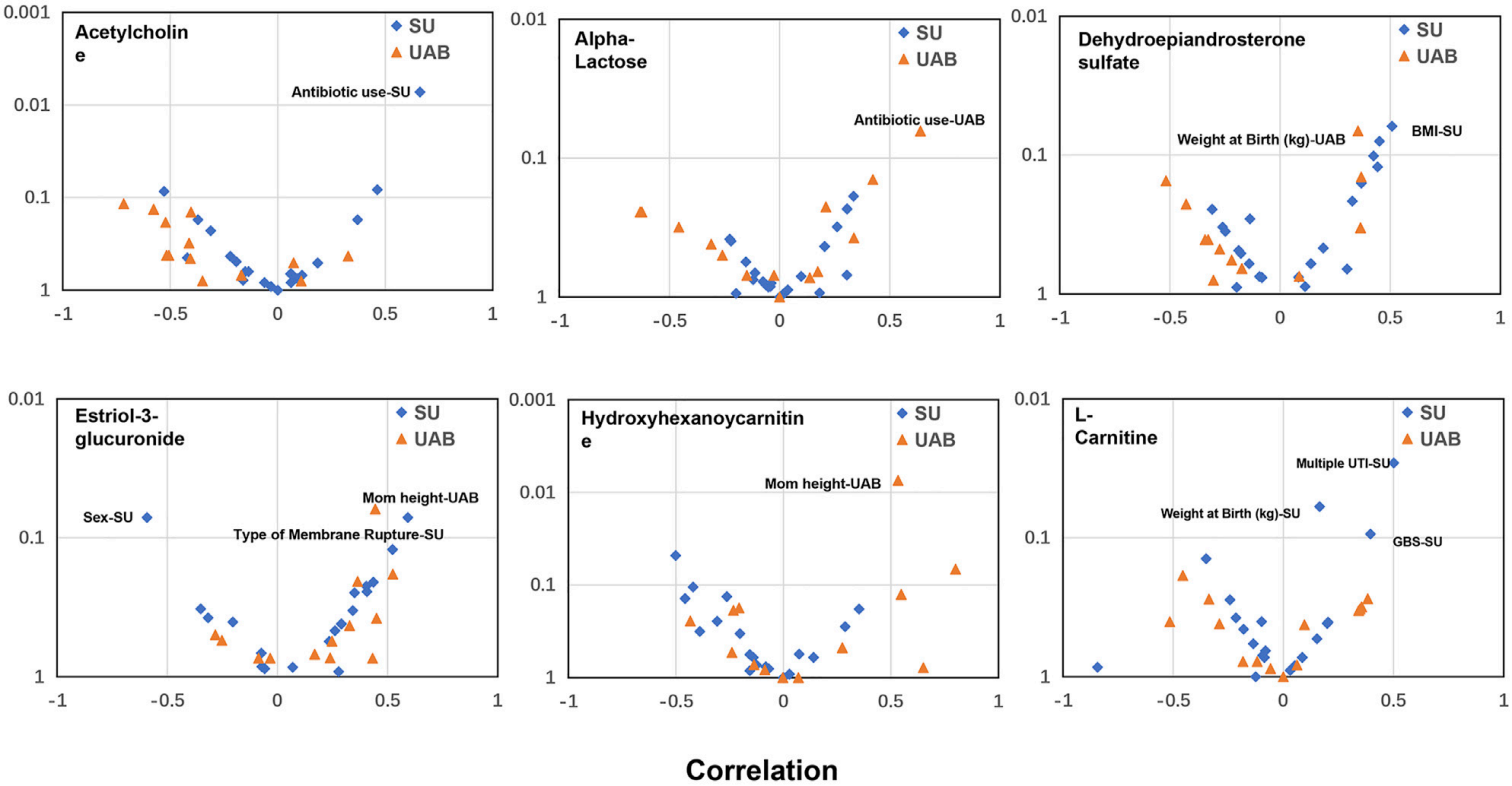
Correlational analysis to reveal significant relationships between metabolite biomarker early gestational expression and the demographics or other determinant variables. *x* axis: Correlation analysis using Pearson method; *Y* axis: *p* value. SU: California cohort; UAB: Alabama cohort. A relationship, with either *p* value <0.05 or Pearson R > 0.5 or < −0.5, is considered to be significant and annotated in the plot. The GBS test is to identify women who carry the bacterium. UTI: urinary tract infection.

A woman’s health in pregnancy is dependent upon the interaction of one’s biologic attributes with one’s social and physical environments. We previously proposed (Stevenson et al., 2021a; Stevenson et al., 2021b) that pregnancy should not be categorized solely in terms of biologic or social determinants if we are to gain a full understanding, even when the concept of social determinants includes a set of broad environmental factors. Understanding the complex interrelationship between biologic and social determinant factors may be facilitated by complex multi-omics analysis with mathematical algorithms. Our correlational analysis of all demographics and other determinant variables (Supplymentary Figure S2) to the individual biomarker gestational expression revealed significant relationships, either shared between the two cohorts or unique to either cohort (Figure 8). Given that the two cohorts have different demographics (Supplymentary Table S1, S2), the results are expected. Our findings suggest that these variables may confound the metabolic analysis in this study, and in the meantime additional analysis of these factors may provide some potential clues to the gestational disorders. For example, the variables, of “antibiotic use” and “weight at birth”, were revealed as significant in both cohorts to different biomarkers. We can hypothesize that antibiotic use is to treat gestational infection which is a risk factor for preterm birth and expect to have impact on the duration of the gestations. “Weight at birth” is an outcome variable of which value can be changed due to gestational disorders, including preterm birth and preeclampsia, resulting early deliveries of low weight babies. Other variables are not shared between the cohorts to have significant correlations with the biomarker gestational expression: sex, BMI, multiple UTI, GBS of the California cohort; mom height of the Alabama cohort. The disparities, revealed by the correlational analysis between the two cohorts, and the their underlying biology, demographics or social determinant network might need additional characterization with larger and independent cohorts from other geographic regions. We believe that the search for solutions to gestational disorders, like many other complex human conditions, will necessarily require a deeper understanding of the complexity of the interactions between biologic and social determinants, using sophisticated multi-omics approaches (Liu et al., 2013; Hao et al., 2020; Sylvester et al., 2020; Huang et al., 2021) and mathematical algorithms linking the various scientific disciplines in a coordinated effort to find the most effective clinical and public health interventions.

One of the strengths of this study is to combine both machine learning and traditional statistical modelling approaches. For future translation of this work into clinical practice to have any practical effect, clinicians expect to understand the underlying predictive features of the models and acknowledge the explainability of individual predictions. We applied the SHAP method to allow the transparency and explainability for our models. Besides global importance, the explainer was used to explain predictions of unseen individual instances to provide insight into which predictors contributed the most in predicting the obtained output.

The study had several limitations. First, the sampling time between the two cohorts was not perfectly matched. Samples in the California cohort were collected as early as week 9, while samples in the Alabama cohort were collected at week 15 or later. Second, some subjects did not have samples collected in every week. Third, LMPs of the study subjects were missing, which, if recorded, could have provided additional reference to fetal growth. Fourth, patterns of some biomarkers and pathways in the maternal urine may have been affected by clinical manifestations of the subjects.

A larger cohort of pregnant women with complete medical records will be assembled to facilitate studies on relationship between the urine metabolic profiling and patient characteristics. Furthermore, this study focused on characterizing the baseline urine metabolic profile of normal pregnancy. Future studies on urine metabolic adaptions in pregnancy with adverse outcomes will provide insight into pathology and pathophysiology assessment.

## Conclusion

In conclusion, we successfully identified a panel of urine metabolites and associated pathways that are highly correlated with pregnancy progression. We also developed models to estimate GA and predict date of delivery, the results of which were comparable with ultrasound measurements. It validated the hypothesis that a “clock” for normal pregnancy progression could be established by maternal urine metabolomics.

## Data Availability

The raw data supporting the conclusion of this article will be made available by the authors, without undue reservation.
