# Supplementary material for: Gestational Dating by Urine Metabolic Profile at High Resolution Weekly Sampling Timepoints: Discovery and Validation
Source: Front Mol Med. 2022 Apr 27;2:844280. doi: 10.3389/fmmed.2022.844280 (PMC11285704; doi:10.3389/fmmed.2022.844280)
Supplement: Supplementary file 1 [file DataSheet1.PDF]

Supplementary Material

Supplemental Table 1. Patient characteristics shared by both California and Alabama cohorts

	California Cohort	Alabama Cohort
**Characteristic**	**Full-term (n=19)**	**Full-term (n=10)**
**Age, mean(SD)**	**32.2 (4.7)**	**26.(4.9)**
**GA @ Del, mean(SD)**	**39.5 (1.2)**	**38.2 (1.2)**
**Days, mean(SD)**	**2.9 (2.1)**	**2.4 (2.2)**
**Type of Membrane Rupture, N(%)**		
**AROM**	**11 (57.9)**	**6 (60)**
**SROM**	**8 (42.1)**	**4 (40)**
**How did labor begin?, N(%)**		
**Augmented**	**3 (15.8)**	**3 (30)**
**Induced**	**5 (26.3)**	**4 (40)**
**Spontaneous**	**9 (47.4)**	**3 (30)**
**SROM w/o contractions**	**1 (5.3)**	**0 (0)**
**NA**	**1 (5.3)**	**0 (0)**
**Mode of delivery, N(%)**		
**Cesarean**	**6 (31.6)**	**0 (0)**
**NSVD**	**12 (63.2)**	**10 (100)**
**Operative Vaginal delivery**	**1 (5.3)**	**0 (0)**
**Presentation @ Del, Vertex/cephalic**	**19 (100)**	**10 (100)**
**Beta blocker Use, No**	**19 (100)**	**10 (100)**
**GBS, N(%)**		
**Negative**	**16 (84.2)**	**7 (70)**
**Positive**	**3 (15.8)**	**3 (30)**
**Blood loss (>500mL for NSVD; >1,000mL for cesarean, N(%)**		
**No**	**17 (89.5)**	**10 (100)**
**Yes**	**2 (10.5)**	**0 (0)**
**Transfusion, N(%)**		

**No**	**18 (94.7)**	**10 (100)**
**Yes**	**1 (5.3)**	**0 (0)**
**Antibiotic use, N(%)**		
**No**	**12 (63.2)**	**7 (70)**
**Yes**	**7 (36.8)**	**3 (30)**
**Preg Type, N(%)**		
**IVF**	**1 (5.3)**	**0 (0)**
**Spontaneous**	**18 (94.7)**	**9 (90)**
**NA**	**0 (0)**	**1 (10)**
**Race, N(%)**		
**White**	**19 (100)**	**0 (0)**
**Black**	**0 (0)**	**10 (100)**
**Ethnicity, Non-hispanic**	**19 (100)**	**10 (100)**
**BMI, median(IQR)**	**21.8 (20.2,24.7)**	**29.8 (26.6,32.5)**
**highest level of education, N(%)**		
**Bachelor/undergraduate degree**	**5 (26.3)**	**0 (0)**
**High School diploma or equivalent (GED)**	**0 (0)**	**3 (30)**
**No high school (8th grade or less)**	**0 (0)**	**1 (10)**
**Post-undergraduate degree**	**12 (63.2)**	**0 (0)**
**Post-undergraduate degree/PhD**	**2 (10.5)**	**0 (0)**
**Some college**	**0 (0)**	**4 (40)**
**Some high school**	**0 (0)**	**2 (20)**
**Smoker, N(%)**		
**No**	**19 (100)**	**8 (80)**
**Yes**	**0 (0)**	**2 (20)**
**Prev Pregs, median(IQR)**	**1 (0,2)**	**3 (1,4)**
**Hx of PTB, N(%)**		
**NO**	**16 (84.2)**	**1 (10)**
**YES**	**3 (15.8)**	**9 (90)**
**Progest Use, N(%)**		
**YES**	**1 (5.3)**	**10 (100)**
**NA**	**18 (94.7)**	**0 (0)**
**Mom Hgt (cm), mean(SD)**	**166.9 (6.2)**	**162.9 (3)**
**Hx of PE, N(%)**		

**No**	**19 (100)**	**0 (0)**
**Chorioamnionitis, N(%)**		
**No**	**17 (89.5)**	**10 (100)**
**Yes**	**2 (10.5)**	**0 (0)**
**Endometritis, N(%)**		
**No**	**18 (94.7)**	**10 (100)**
**Yes**	**1 (5.3)**	**0 (0)**
**Autoimmune Disorder, N(%)**		
**No**	**18 (94.7)**	**10 (100)**
**Yes**	**1 (5.3)**	**0 (0)**
**Multiple UTI, N(%)**		
**No**	**17 (89.5)**	**10 (100)**
**Yes**	**2 (10.5)**	**0 (0)**
**Psychiatric Dx (depression), N(%)**		
**No**	**17 (89.5)**	**9 (90)**
**Yes**	**2 (10.5)**	**1 (10)**
**Thyroid Dx, N(%)**		
**No**	**18 (94.7)**	**10 (100)**
**Yes**	**1 (5.3)**	**0 (0)**
**Asthma, N(%)**		
**No**	**18 (94.7)**	**9 (90)**
**Yes**	**1 (5.3)**	**1 (10)**
**Cancer, N(%)**		
**No**	**18 (94.7)**	**10 (100)**
**Yes**	**1 (5.3)**	**0 (0)**
**Epilepsy, N(%)**		
**No**	**19 (100)**	**9 (90)**
**Yes**	**0 (0)**	**1 (10)**
**Breast Dx, No**	**19 (100)**	**10 (100)**
**Gynecol surgery, N(%)**		
**No**	**14 (73.7)**	**10 (100)**
**Yes**	**5 (26.3)**	**0 (0)**
**HTN, N(%)**		
**No**	**19 (100)**	**9 (90)**

**Yes**	**0 (0)**	**1 (10)**
**Gest HTN, N(%)**		
**Mild gHTN**	**1 (5.3)**	**0 (0)**
**No**	**11 (57.9)**	**0 (0)**
**NA**	**7 (36.8)**	**10 (100)**
**EtOH, N(%)**		
**No**	**18 (94.7)**	**10 (100)**
**Yes**	**1 (5.3)**	**0 (0)**
**Recreational Drug Use, N(%)**		
**No**	**19 (100)**	**9 (90)**
**Yes**	**0 (0)**	**1 (10)**
**Sex, N(%)**	**0 (0)**	
**Female**	**8 (42.1)**	**5 (50)**
**Male**	**11 (57.9)**	**5 (50)**
**Wgt at Birth (kg), mean(SD)**	**3.4 (0.3)**	**3.1 (0.5)**
**Length at birth (cm), median(IQR)**	**51 (50,53)**	**49.5 (46.8,50.6)**

Supplemental Table 2A. Patient characteristics of California cohort.

Characteristic	California Cohort
	Full-term (n=19)
Age, mean(SD)	32.2 (4.7)
GA @ Del, mean(SD)	39.5 (1.2)
Days, mean(SD)	2.9 (2.1)
Type of Membrane Rupture, N(%)	
AROM	11 (57.9)
SROM	8 (42.1)
How did labor begin?, N(%)	
Augmented	3 (15.8)
Induced	5 (26.3)
Spontaneous	9 (47.4)
SROM w/o contractions	1 (5.3)
NA	1 (5.3)
Mode of delivery, N(%)	
Cesarean	6 (31.6)
NSVD	12 (63.2)
Operative Vaginal delivery	1 (5.3)
Presentation @ Del, Vertex/cephalic	19 (100)
Beta blocker Use, No	19 (100)
GBS, N(%)	
Negative	16 (84.2)
Positive	3 (15.8)
Blood loss (>500mL for NSVD; >1,000mL for cesarean, N(%)	
No	17 (89.5)
Yes	2 (10.5)
Transfusion, N(%)	
No	18 (94.7)
Yes	1 (5.3)
Antibiotic use, N(%)	
No	12 (63.2)

**Yes**	**7 (36.8)**
**Preg Type, N(%)**	
**IVF**	**1 (5.3)**
**Spontaneous**	**18 (94.7)**
**Race, N(%)**	
**White**	**19 (100)**
**Ethnicity, Non-hispanic**	**19 (100)**
**BMI, median(IQR)**	**21.8 (20.2,24.7)**
**highest level of education, N(%)**	
**Bachelor/undergraduate degree**	**5 (26.3)**
**High School diploma or equivalent (GED)**	**0 (0)**
**No high school (8th grade or less)**	**0 (0)**
**Post-undergraduate degree**	**12 (63.2)**
**Post-undergraduate degree/PhD**	**2 (10.5)**
**Smoker, N(%)**	
**No**	**19 (100)**
**Prev Pregs, median(IQR)**	**1 (0,2)**
**Hx of PTB, N(%)**	
**NO**	**16 (84.2)**
**YES**	**3 (15.8)**
**Progest Use, N(%)**	
**NA**	**18 (94.7)**
**YES**	**1 (5.3)**
**Mom Hgt (cm), mean(SD)**	**166.9 (6.2)**
**Mom Wgt (kg), median(IQR)**	**61 (57,66.7)**
**Hx of PE, N(%)**	
**No**	**19 (100)**
**Chorioamnionitis, N(%)**	
**No**	**17 (89.5)**
**Yes**	**2 (10.5)**
**Endometritis, N(%)**	
**No**	**18 (94.7)**
**Yes**	**1 (5.3)**

**Autoimmune Disorder, N(%)**	
**No**	**18 (94.7)**
**Yes**	**1 (5.3)**
**Multiple UTI, N(%)**	
**No**	**17 (89.5)**
**Yes**	**2 (10.5)**
**Psychiatric Dx (depression), N(%)**	
**No**	**17 (89.5)**
**Yes**	**2 (10.5)**
**Thyroid Dx, N(%)**	
**No**	**18 (94.7)**
**Yes**	**1 (5.3)**
**Asthma, N(%)**	
**No**	**18 (94.7)**
**Yes**	**1 (5.3)**
**Cancer, N(%)**	
**No**	**18 (94.7)**
**Yes**	**1 (5.3)**
**Epilepsy, N(%)**	
**No**	**19 (100)**
**Yes**	**0 (0)**
**Breast Dx, No**	**19 (100)**
**Gynecol surgery, N(%)**	
**No**	**14 (73.7)**
**Yes**	**5 (26.3)**
**HTN, N(%)**	
**No**	**19 (100)**
**Gest HTN, N(%)**	
**Mild gHTN**	**1 (5.3)**
**No**	**11 (57.9)**
**NA**	**7 (36.8)**
**EtOH, N(%)**	
**No**	**18 (94.7)**
**Yes**	**1 (5.3)**

**Recreational Drug Use, N(%)**	
**No**	**19 (100)**
**Sex, N(%)**	**0 (0)**
**Female**	**8 (42.1)**
**Male**	**11 (57.9)**
**Wgt at Birth (kg), mean(SD)**	**3.4 (0.3)**
**Length at birth (cm), median(IQR)**	**51 (50,53)**

Supplemental Table 2B. Patient characteristics of Alabama cohort.

Characteristic	Alabama Cohort Full-term (n=10)
Age, mean(SD)	26.1 (4.9)
GA @ Del, mean(SD)	38.2 (1.2)
Days, mean(SD)	2.4 (2.2)
Type of Membrane Rupture, N(%)	
AROM	6 (60)
SROM	4 (40)
How did labor begin?, N(%)	
Augmented	3 (30)
Induced	4 (40)
Spontaneous	0 (0)
SROM w/o contractions	3 (30)
NA	0 (0)
Mode of delivery, N(%)	
Cesarean	0 (0)
NSVD	10 (100)
Operative Vaginal delivery	0 (0)
Presentation @ Del, Vertex/cephalic	10 (100)
Beta blocker Use, No	10 (100)
GBS, N(%)	
Negative	7 (70)
Positive	3 (30)
Blood loss (>500mL for NSVD; >1,000mL for cesarean, N(%)	
No	10 (100)
Yes	0 (0)
Transfusion, N(%)	
No	10 (100)
Yes	0 (0)

**Antibiotic use, N(%)**	
**No**	**7 (70)**
**Yes**	**3 (30)**
**Preg Type, N(%)**	
**Spontaneous**	**9 (90)**
**NA**	**1 (10)**
**Race, N(%)**	
**Black**	**10 (100)**
**Ethnicity, Non-hispanic**	**10 (100)**
**BMI, median(IQR)**	**29.8 (26.6,32.5)**
**highest level of education, N(%)**	
**High School diploma or equivalent (GED)**	**3 (30)**
**No high school (8th grade or less)**	**1 (10)**
**Some college**	**4 (40)**
**Some high school**	**2 (20)**
**Smoker, N(%)**	
**No**	**8 (80)**
**Yes**	**2 (20)**
**Prev Pregs, median(IQR)**	**3 (1,4)**
**Hx of PTB, N(%)**	
**NO**	**1 (10)**
**YES**	**9 (90)**
**Progest Use, N(%)**	
**YES**	**10 (100)**
**Mom Hgt (cm), mean(SD)**	**162.9 (3)**
**Mom Wgt (kg), median(IQR)**	**78.8 (67.5,88)**
**Hx of PE, N(%)**	
**NA**	**10 (100)**
**Chorioamnionitis, N(%)**	

**No**	**10 (100)**
**Endometritis, N(%)**	
**No**	**10 (100)**
**Autoimmune Disorder, N(%)**	
**No**	**10 (100)**
**Multiple UTI, N(%)**	
**No**	**10 (100)**
**Psychiatric Dx (depression), N(%)**	
**No**	**9 (90)**
**Yes**	**1 (10)**
**Thyroid Dx, N(%)**	
**No**	**10 (100)**
**Asthma, N(%)**	
**No**	**9 (90)**
**Yes**	**1 (10)**
**Cancer, N(%)**	
**No**	**10 (100)**
**Epilepsy, N(%)**	
**No**	**9 (90)**
**Yes**	**1 (10)**
**Breast Dx, No**	**10 (100)**
**Gynecol surgery, N(%)**	
**No**	**10 (100)**
**HTN, N(%)**	
**No**	**9 (90)**
**Yes**	**1 (10)**
**Gest HTN, N(%)**	
**NA**	**10 (100)**
**EtOH, N(%)**	
**No**	**10 (100)**
**Recreational Drug Use, N(%)**	
**No**	**9 (90)**
**Yes**	**1 (10)**
**Sex, N(%)**	**0 (0)**

**Female**	**5 (50)**
**Male**	**5 (50)**
**Wgt at Birth (kg), mean(SD)**	**3.1 (0.5)**
**Length at birth (cm), median(IQR)**	**49.5 (46.8,50.6)**

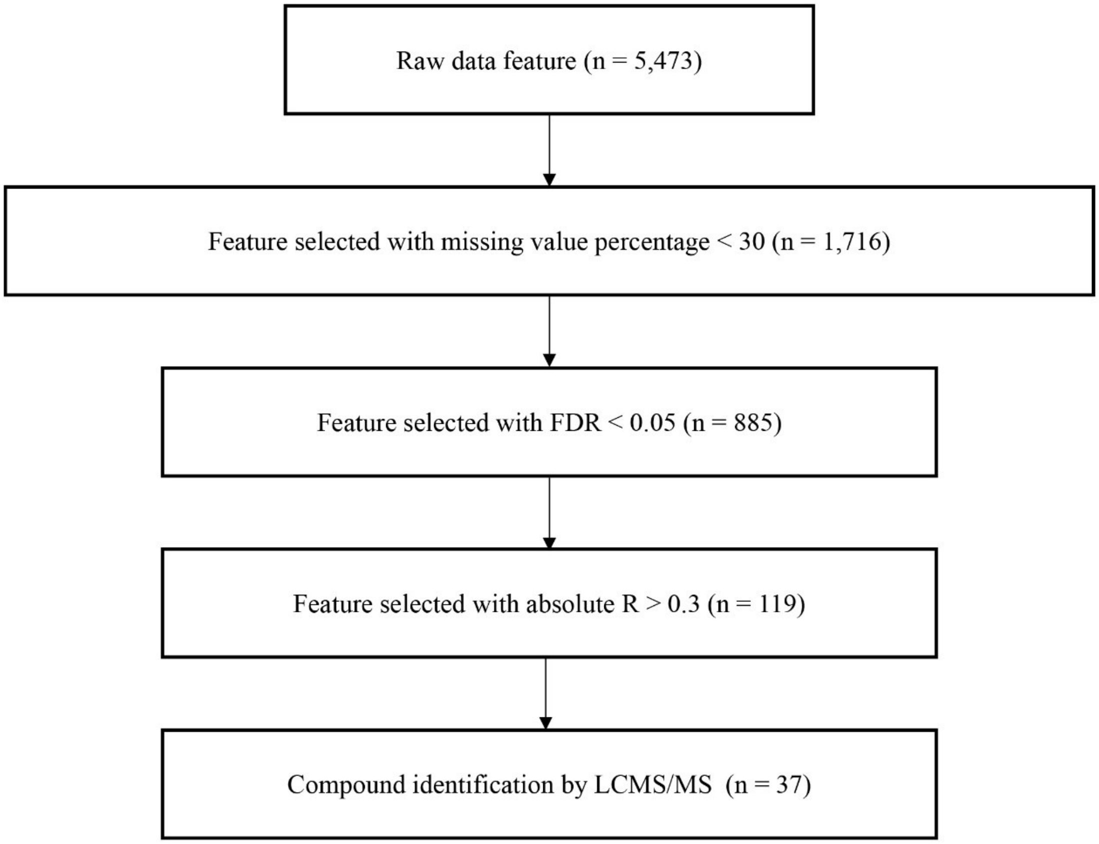

Supplementary Figure 1. Data preprocessing and feature reduction. A total of 5,473 metabolic features were identified by LC-MS-based untargeted metabolomics. Of these, 1,716 features were selected with missing value percentage less than 30% across all samples. 119 features were selected based on FDR and Pearson's screening. Finally, 37 compounds were annotated.

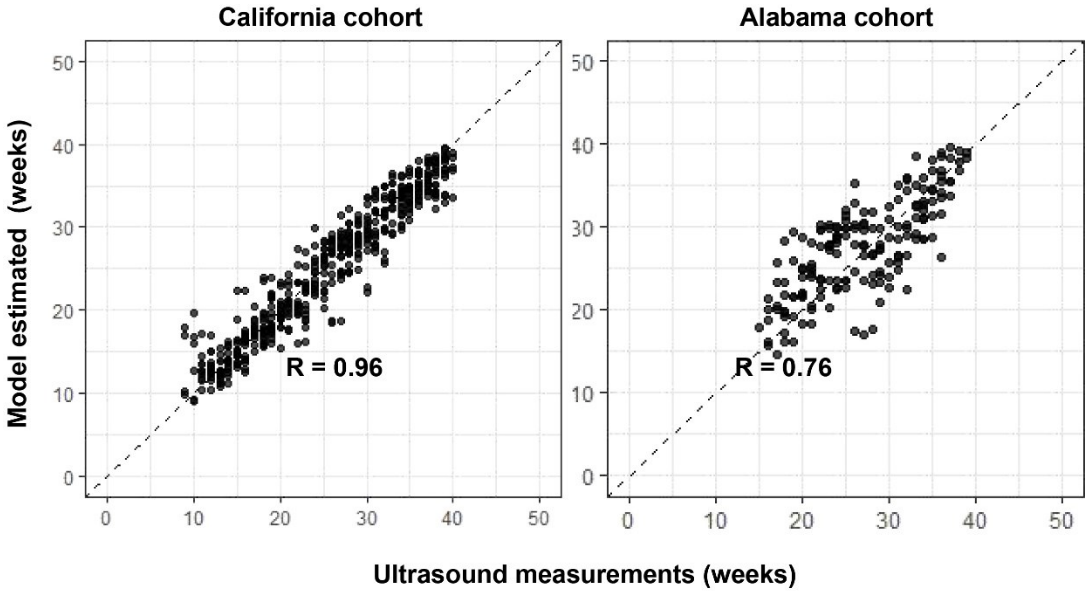

Supplementary Figure 2. A pathway-based model to estimate GA. X axis: GA measured by ultrasound at the first trimester. Y axis: model estimation. R: Pearson's correlation coefficient. Left: California cohort; Right: Alabama cohort.

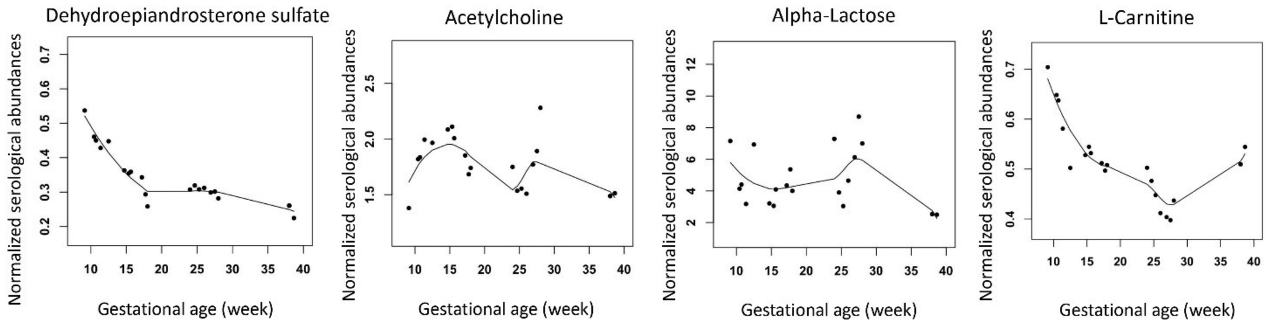

Suppmementary Figure 3. Profile of dehydroepiandrosterone sulfate, acetylcholine, α -lactose, and L-carnitine in maternal serum over the course of gestation on the California cohort. Loess regression lines were plotted.

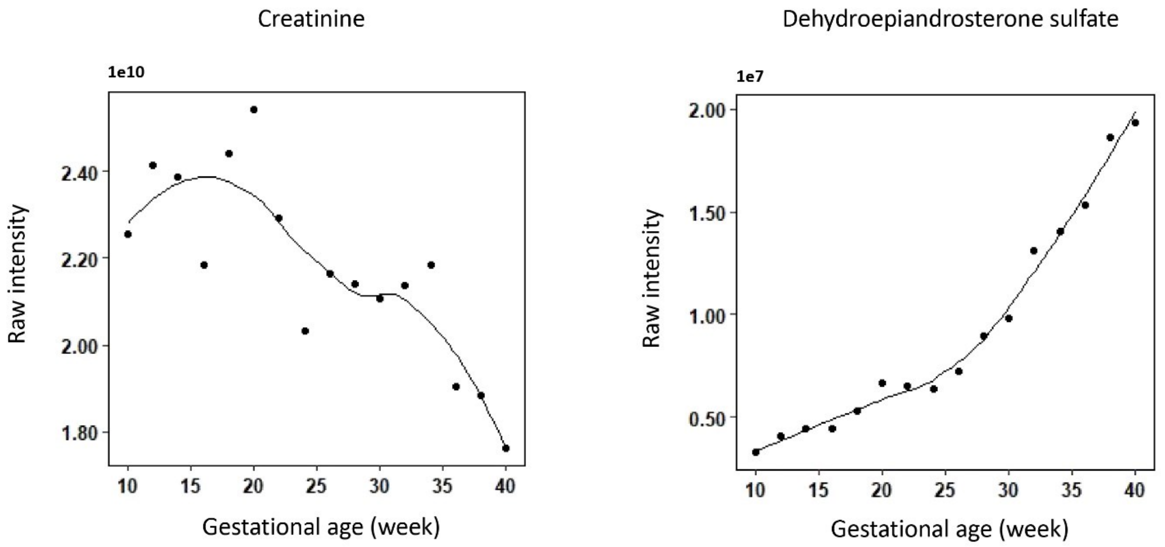

Supplementary Figure 4. Intensity of creatinine (left) and dehydroepiandrosterone sulfate (right) in urine over the course of gestation on California cohort. Mean ± standard error of the mean at each time point was plotted.
